# A systemic review and meta-analysis comparing the ability of diagnostic of the third heart sound and left ventricular ejection fraction in heart failure

**DOI:** 10.3389/fcvm.2022.918051

**Published:** 2022-10-06

**Authors:** Lin Dao, Min Huang, Xinghong Lin, Liuyin Li, Xixi Feng, Changyou Wei, Mingjin Guo, Yifan Yang, Fan Xu, Xiechuan Weng

**Affiliations:** ^1^Department of Clinic Medicine, Chengdu Medical College, Chengdu, Sichuan, China; ^2^Department of Physiology, Chengdu Medical College, Chengdu, Sichuan, China; ^3^Department of Chinese Medicine Heart Disease, Zigong City First People’s Hospital, Zigong, Sichuan, China; ^4^Department of Public Health, Chengdu Medical College, Chengdu, Sichuan, China; ^5^Department of Vascular Surgery, The Affiliated Hospital of Qingdao University, Qingdao, China; ^6^Department of Pain Medicine, Peking University Peoples Hospital, Beijing, China; ^7^Department of Neuroscience, Beijing Institute of Basic Medical Sciences, Beijing, China

**Keywords:** acute heart failure, LVEF, meta-analysis, third heart sound, heart failure

## Abstract

**Objective:**

This study aimed to compare the sensitivity and specificity of diagnosis between the third heart sound (S3) and left ventricular ejection fraction (LVEF) in heart failure (HF).

**Methods:**

Relevant studies were searched in PubMed, SinoMed, China National Knowledge Infrastructure, and the Cochrane Trial Register until February 20, 2022. The sensitivity, specificity, likelihood ratio (LR), and diagnostic odds ratio (DOR) were pooled. The symmetric receiver operator characteristic curve (SROC) and Fagan’s nomogram were drawn. The source of heterogeneity was explored by meta-regression and subgroup analysis.

**Results:**

A total of 19 studies, involving 5,614 participants, were included. The combined sensitivity of S3 was 0.23 [95% confidence interval (CI) (0.15–0.33), specificity was 0.94 [95% CI (0.82–0.98)], area under the SROC curve was 0.49, and the DOR was 4.55; while the sensitivity of LVEF was 0.70 [95% CI (0.53–0.83)], specificity was 0.79 [95% CI (0.75–0.82)], area under the SROC curve was 0.79, and the DOR was 8.64. No publication bias was detected in Deeks’ funnel plot. The prospective design, partial verification bias, and blind contributed to the heterogeneity in specificity, while adequate description of study participants contributed to the heterogeneity in sensitivity. In Fagan’s nomogram, the post-test probability was 48% when the pre-test probability was set as 20%, while in LVEF, the post-test probability was 45% when the pre-test probability was set as 20%.

**Conclusion:**

The use of S3 alone presented lower sensitivity in diagnosing HF compared with LVEF, whereas it was useful in early pathological assessment.

## Introduction

Heart failure (HF) is a pathological process during the pumping of blood in the heart. The cardiac output becomes insufficient to fully meet the needs of body metabolism (1–3). Currently, the 5 year mortality rate for HF has remained around 50% (4). The traditional diagnosis of HF relies mainly on the history and physical examination; the clinical diagnostic methods for HF include bio-standard object examination, electrocardiogram, echocardiogram, cardiac magnetic resonance imaging, and invasive hemodynamic monitoring (5). The ratio of stroke volume to ventricular end-diastolic volume is called the ejection fraction. The ejection fraction accurately reflects the pumping function of the heart, which is important for the early detection of cardiac pumping dysfunction. Left ventricular ejection fraction (LVEF) is an important diagnostic index of HF and an important basis for its classification (5).

The non-invasive detection method has been used for the effective diagnosis of early HF without organic heart disease or clinical symptoms (6). As routine cardiac physical examination, heart sound auscultation helps in cardiac function evaluation and initial screening of cardiac structure abnormalities, and has important value for the early diagnosis of cardiovascular diseases (7). Heart sound signals, especially the third heart sound (S3) signals, are associated with increased left ventricular end-diastolic pressure, and considered ideal confirmatory markers (8). Recently, the application of heart sound analysis in the diagnosis and classification of HF has emerged gradually. However, the value of diagnosis using heart sounds in HF remains controversial. Therefore, this study aimed to compare the sensitivity and specificity of diagnosis between S3 and LVEF in HF.

## Methods

### Search strategy

Two reviewers (DL and LXJ) searched the PubMed, Embase, Cochrane Library, China National Knowledge Infrastructure, and Wan Fang databases up to February 2022 independently. The search terms were as follows: #1 TS = (“HF, Diastolic” OR “HF, Systolic” OR “Ventricular Dysfunction, Right” OR “Ventricular Dysfunction, Left”); #2 TS = (the S3 OR Heart Auscultation OR Heart Sounds OR Sounds, Heart OR Cardiac Sounds OR Cardiac Sound OR Sound, and Cardiac OR Sounds, Cardiac); #3 DT = (Clinical Trial OR Article); #4 DOP = (1971-01-01/2022-2-22); #5 #1 AND #2 AND #3 AND #4.

### Inclusion and exclusion criteria

The inclusion criteria were as follows: (1) randomized controlled experiments using patients with HF as the experimental group and healthy people or patients with benign disease as the control group; (2) well-defined patients with HF included as study participants; (3) diagnostic tests including S3 or/and LVEF; (4) number of true-positive (TP) cases, false-negative (FN) cases, false-positive (FP) cases, and true-negative (TN) cases obtained directly or calculated through the literature; (5) age, sex, and race not considered; and (6) studies published in any language. The exclusion criteria were as follows: (1) animal studies; (2) non-case-control trials; (3) studies with incomplete or no experimental data, duplicate published literature, reviews, and abstracts; (4) poor equilibrium between groups and different baselines, and the two groups not compared with the literature; and (5) no described diagnostic tests.

### Data extraction

Two authors (LD and XL) independently extracted the demographic data and treatment information; the third author (MH) was consulted when disagreement occurred. The baseline information extracted from 23 studies contained the first author’s name, year of publication, title, design type, study participants (number, age, and male/female ratio), disease degree, and length of the disease. The primary outcomes included FN, TN, TP, and FP with S3 and LVEF.

### Statistical analysis

A meta-analysis was performed with Stata 15.0 software (Stata Corp., College Station, TX, USA). The combined sensitivity, specificity, positive/negative likelihood ratio (PLR/NLR), and diagnostic odds ratio (DOR) were calculated using the bivariate model. The total diagnostic accuracy was estimated by drawing the symmetric receiver operator characteristic curve (SROC). Post-test probability was used to determine whether the probability of diagnosis increased or reduced compared with pre-test probability, which was estimated from routine data, practice data, or clinical judgment. Heterogeneity was assessed using Cochrane’s *Q* statistics (chi-square) or inverse variance (*I*^2^). *I*^2^ < 50% and *P* > 0.1 indicated that these studies could be considered homogeneous using a fixed-effects model. If *I*^2^ ≥ 50% and *P* < 0.10, the random-effects model was used for meta-analysis. A *P*-value < 0.05 indicated a significant difference.

## Results

### Flow chart and study quality

**FIGURE 1:**
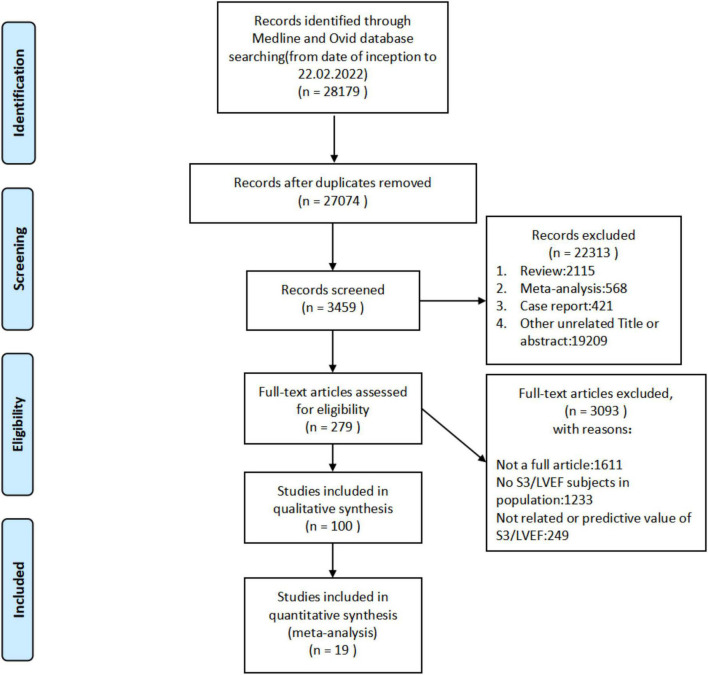
Literature screening process of the meta-analysis.

A total of 28,179 studies (including documents, reviews, animal experiments, case reports, and repeated studies) were retrieved from each database. After removing 27,074 duplicate records, 279 relevant studies were included. Among these studies, 2,115 were excluded for being reviews, meta-analyses, or case reports, while 19,209 studies did not have related titles and abstracts. The full text of the remaining 279 studies was read and 3,093 studies were removed after reading the full text due to incomplete data. The remaining 19 studies were extracted from the corresponding data according to the data extraction requirements. Twelve studies used S3, and seven used LVEF. The literature screening process is shown in Figure 1. The basic characteristics and inclusion and exclusion criteria of each study included are shown in Table 1 and Supplementary Table 1.

**TABLE 1:** Basic characteristics of enrolled studies.

References	Study	Region	N	Age (mean ± SD)	Sex (male%)
Dao et al. (9)	Retrospective	America	250	CHF (*n* = 250): 63 ± 0.86;	CHF: male/female = 94:6
Knudsen et al. (10)	Retrospective	America	880	HF (*n* = 447): 64 ± 16; No-HF (*n* = 433): 64 ± 16	HF: 482 (55)
Knudsen et al. (11)	Retrospective	Norway	155	CHF (*n* = 155): men (*n* = 69): 74 (66–79); women (*n* = 86): 78 (71–84)	Men: 69 (44.5)
Zhang (12)	Prospective	China	78	CHF (*n* = 42): 63 ± 12; non-CHF (*n* = 36): 54 ± 12	CHF: 29 (69.0); non-CHF: 21 (58.3)
Collins et al. (13)	Prospective	America	343	Primary HF (*n* = 133): 69 (30–97); secondary HF (*n* = 60): 68 (39–93); non-HF (*n* = 150): 55 (20–95)	Primary HF: 70 (52.6); secondary HF: 26 (43.3); non-HF: 63 (42.0)
Collins et al. (14)	Prospective	America	1,076	ADHF (*n* = 413): 68 (40–95); no-ADHF (*n* = 506): 59.5 (40–95)	ADHF: 246 (59.6) No-ADHF: 255 (50.4)
Wang et al. (15)	Retrospective	China	292	HBP (*n* = 94): 54 ± 10; HFREF (*n* = 89): 73 ± 13; HFNEF (*n* = 109): 77 ± 10	HBP: 46 (49); HFREF: 66 (74); HFNEF: 46 (42)
Dieplinger et al. (16)	Prospective	Austria	251	CHF (*n* = 137): 76 (69–82); non-CHF (*n* = 114): 69 (58–78)	CHF: 128 (93); non-CHF: 106 (93)
Miller et al. (17)	Prospective	USA	89	AHF (*n* = 35): 72 ± 10; non-AHF (*n* = 54): 65 ± 10	AHF: 24 (69); non-AHF; 28 (52)
Wang et al. (18)	Retrospective	China	127	HF: 72 ± 13 (36–97)	HF: 91 (71.7)
Logeart et al. (19)	Prospective	America	163	CHF (*n* = 115): 68.3 ± 14.7; non-CHF (*n* = 48): 65.1 ± 15.1	Male/female CHF (*n* = 115): 80/35; non-CHF (*n* = 48): 29/19
Steg et al. (20)	Prospective	America	709	CHF (*n* = 492): 68.5 ± 14.1; non-CHF (*n* = 217): 61.6 ± 14.8	Male/female CHF (*n* = 492): 217/275 non-CHF (*n* = 217): 90/127
Nazerian et al. (21)	Prospective	Italy	145	aLVHF (*n* = 64): 8 ± 8; Others (*n* = 81): 75 ± 12	aLVHF (female): 33 (54); others (female): 41 (51)
Anderson et al. (22)	Prospective	America	101	ADHF (*n* = 44): 63 (53–91); non-ADHF (*n* = 57): 62 (52–88)	ADHF: 25 (56); non-ADHF: 27 (47)
Kajimoto et al. (23)	Prospective	Japan	90	AHFS group (*n* = 53): 77.7 ± 10.3; pulmonary group (*n* = 37):78.6 ± 9.2	AHFS group (female): 29 (54.7); pulmonary group (female): 16 (43.2)
Hu (24)	Prospective	China	100	CHF (*n* = 50): 63.55 ± 2.4; non-CHF (*n* = 50): 63.5 ± 2.35	CHF: 33 (66); Non-CHF: 35 (70)
Jiang (25)	Prospective	China	60	CHF (*n* = 48): 59.14 ± 6.82; non-CHF (*n* = 12): 59.14 ± 6.82	CHF: 37 (61.7) non-CHF: NA
Logeart et al. (19)	Prospective	America	163	CHF (*n* = 115): 68.3 ± 14.7; non-CHF (*n* = 48): 65.1 ± 15.1	CHF: 80 (52.7); non-CHF: 29 (17.8)
Steg et al. (20)	Prospective	America	709	CHF (*n* = 492): 68.5 ± 14.1; No CHF (*n* = 217): 61.6 ± 14.8	CHF: 217 (44.1); No CHF: 90 (41.5)

NA, not available from original study paper or supplementary or registration information; ED, emergency department.

### Third heart sound against heart failure

**FIGURE 2:**
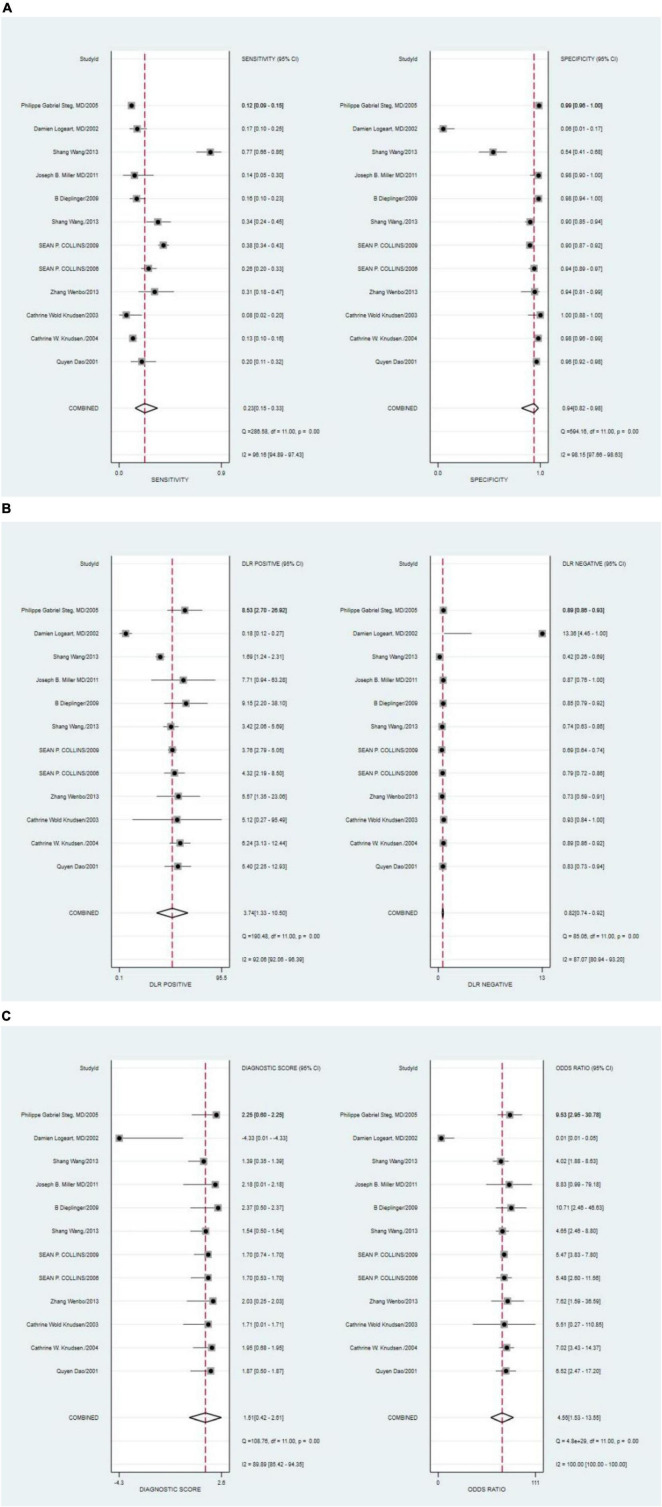
**(A)** Forest plot of sensitivity and specificity of third heart sound (S3) in the diagnosis of heart failure (HF). **(B)** Forest plot of DLR positives and negatives of HF. **(C)** Forest plot of the diagnostic odds ratio (DOR) of S3 in the diagnosis of HF.

The combined sensitivity of S3 in HF was 0.23 [95% CI (0.15–0.33)], specificity was 0.94 [95% CI (0.82–0.98)], PLR was 3.74 95% CI (1.33–10.50)], NLR was 0.82 [95% CI (0.74–0.92)], and DOR was 4.55, indicating that S3 had a medium value in the screening of HF. The random-effects model was used when the heterogeneity was *I*^2^ > 50%. The details of the combined sensitivity and specificity forest are shown in Figure 2A; the combined likelihood ratio (LR) forest is shown in Figure 2B; and the combined diagnosis ratio forest is shown in Figure 2C.

### Publication bias and heterogeneity

**FIGURE 3:**
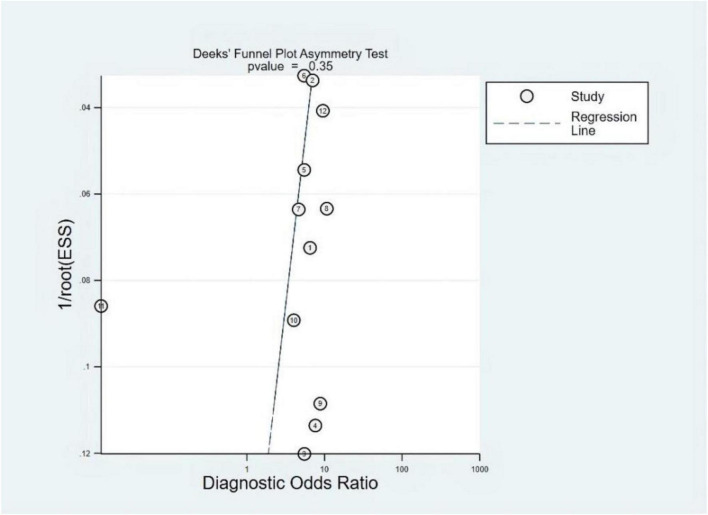
Deeks’ funnel plot.

**FIGURE 4:**
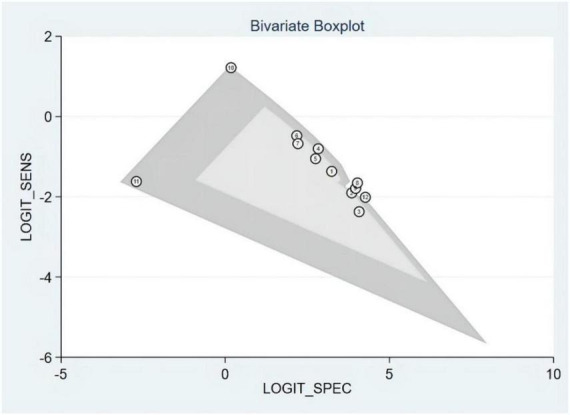
Bivariate boxplot.

The Deeks’ funnel plots were used to assess potential publication bias in detecting HF with S3. As shown in Figure 3, no publication bias existed, with a *P*-value of 0.35. The bivariate boxplot showed that three studies were out of the circles, indicating heterogeneity between included studies, as shown in Figure 4.

### Threshold effect

**FIGURE 5:**
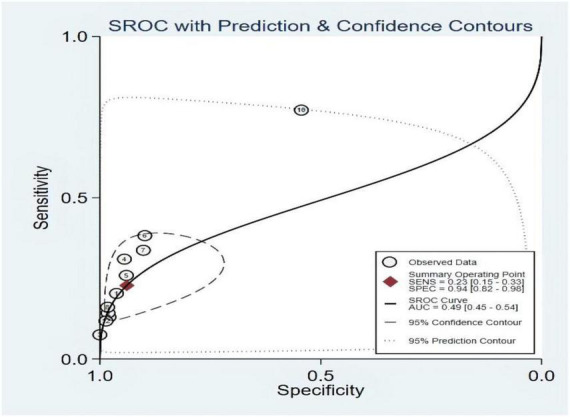
Summary receiver operating characteristic of third heart sound (S3).

The symmetric receiver operator characteristic curve curve plane test was used for the threshold effect. No typical “shoulder arm” was found, indicating no threshold effect. A moderate predictive value could be concluded by the value of the area under the SROC curve (AUC), which was 0.49 [95% CI (0.45–0.54)], as shown in Figure 5.

### Pre-test probability, likelihood ratio, and post-test probability

**FIGURE 6:**
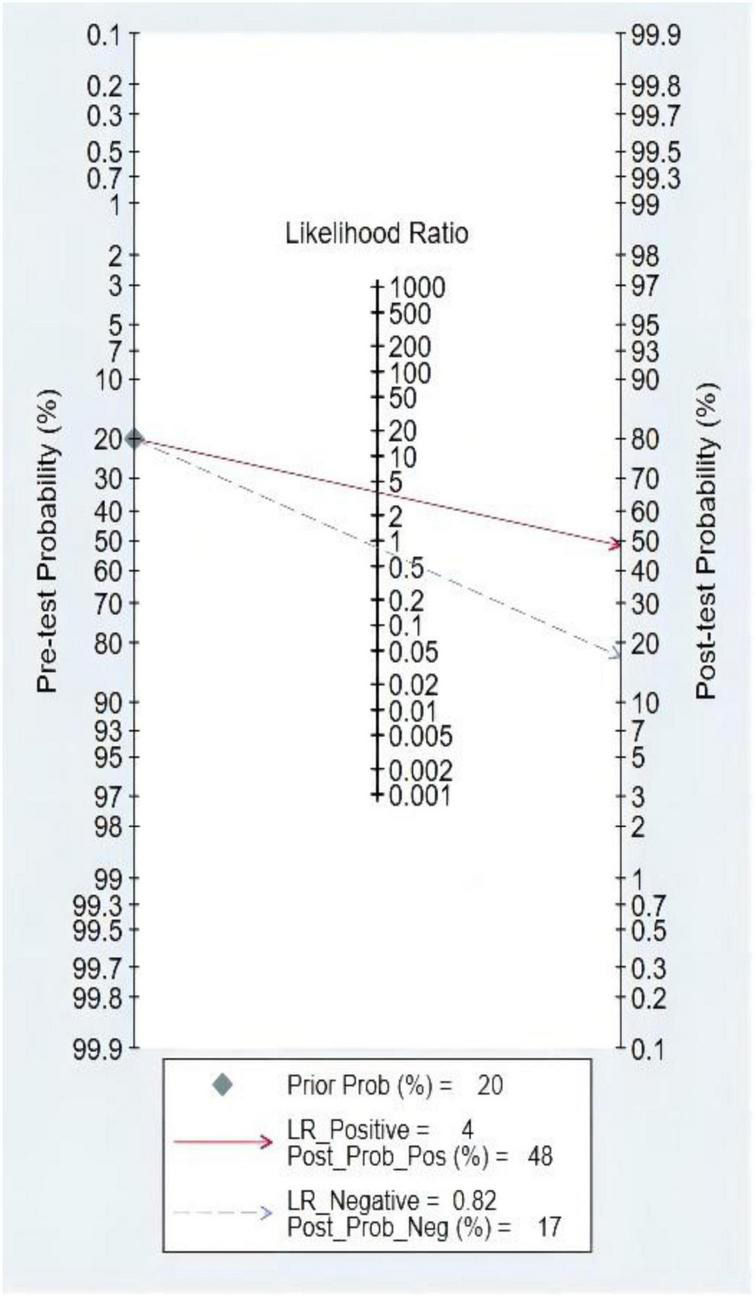
Fagan diagram of third heart sound (S3) in the diagnosis of heart failure (HF).

The Fagan graph was plotted to show the relationship among the prior probability, the LR, and the posterior probability. The pre-test probability was 20% and the post-test probability of HF was 48%. In addition, the positive likelihood ratio (LRP) was <10 (LRP = 4) and the negative likelihood ratio (LRN) was >0.1 (LRN = 0.82), indicating that the diagnosis could neither be confirmed nor excluded. The predictive value of S3 in HF was limited, as shown in Figure 6.

### Meta-regression and subgroup analysis

**FIGURE 7:**
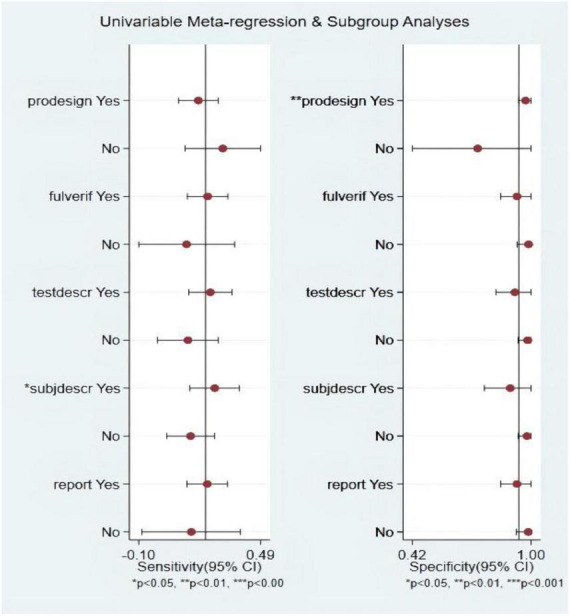
Multiple univariate meta-regression and subgroup analysis. Prospective design: prodesign; fulverif: partial verification bias; subjdescr: adequate description of study participants; brdspect: broad spectrum of disease.

Among the S3 studies, the factors that might affect the heterogeneity, including prospective design (prodesign), partial verification bias (fulverif), an adequate description of study participants (subjdescr), report, a broad spectrum of diseases (brdspect), and whether the test results were evaluated by a blind method, were evaluated. The meta-regression analysis of the aforementioned factors revealed that the sources of heterogeneity of sensitivity were statistically related to subjdescr and the sources of heterogeneity of specificity were related to prodesign, as shown in Figure 7.

### Left ventricular ejection fraction against heart failure

**FIGURE 8:**
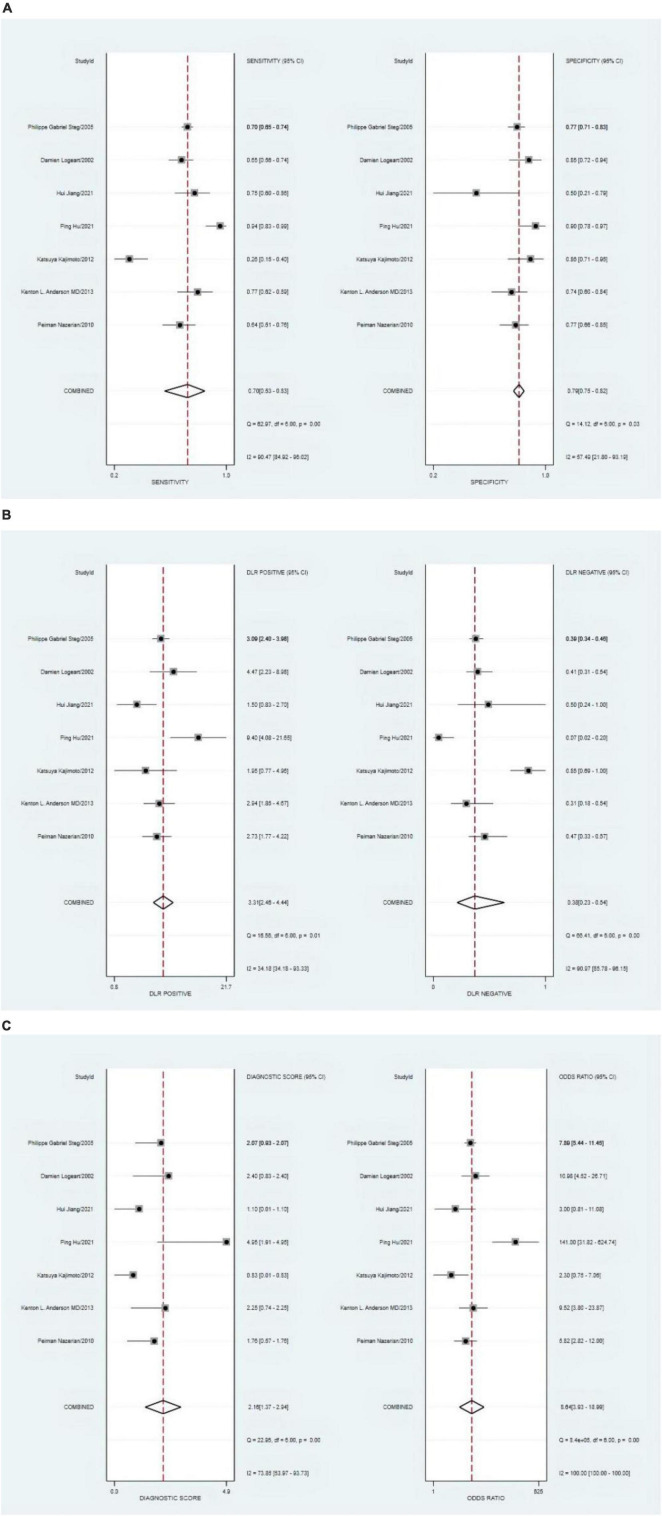
**(A)** Forest plot of sensitivity and specificity of left ventricular ejection fraction (LVEF) in diagnosing heart failure (HF). **(B)** Forest plot of DLR positives and negatives of HF. **(C)** Forest plot of the diagnostic odds ratio (DOR) of LVEF in diagnosing HF.

The combined sensitivity was 0.70 (95% CI, 0.53–0.83), specificity was 0.79 (95% CI, 0.75–0.82), PLR was 3.31 (95% CI, 2.46–4.44), NLR was 0.38 (95% CI, 0.23–0.64), and DOR was 8.64, indicating that the LVEF had a medium value in the screening of HF. The heterogeneity was *I*^2^ > 50%; therefore, the random model was used, as shown in Figure 8.

### Publication bias and heterogeneity

**FIGURE 9:**
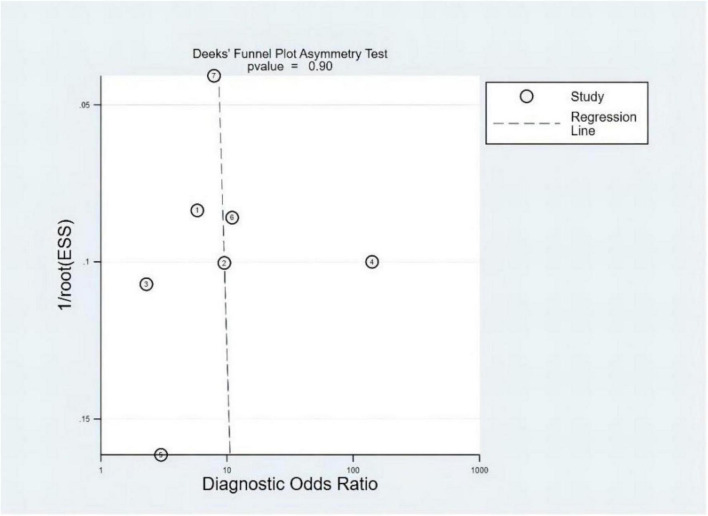
Deeks’ funnel plot.

**FIGURE 10:**
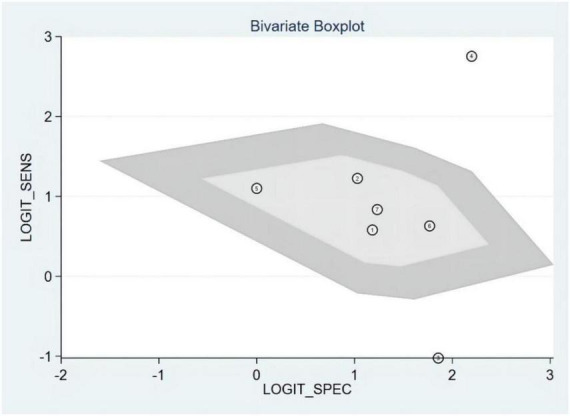
Bivariate boxplot.

The *P-*value of Deeks’ funnel plots asymmetry test was 0.90 (*P* > 0.05). As shown in Figure 9, no evidence of publication bias was found. It demonstrated that three sets of data were out of the circles, indicating heterogeneity between included studies. The details are shown in Figure 10.

### Threshold effect

**FIGURE 11:**
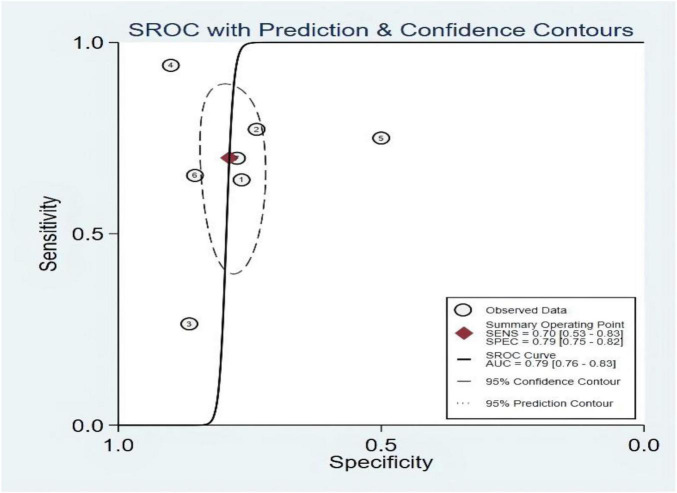
Summary receiver operating characteristic of left ventricular ejection fraction (LVEF).

The threshold effect was assessed using the SROC curve plane test. As no typical “shoulder arm” was found, no threshold effect was observed. A moderate predictive value could be concluded using the value of the AUC, which was 0.79 (95% CI, 0.75–0.83). The details are shown in Figure 11.

### Pre-test probability, likelihood ratio, and post-test probability

**FIGURE 12:**
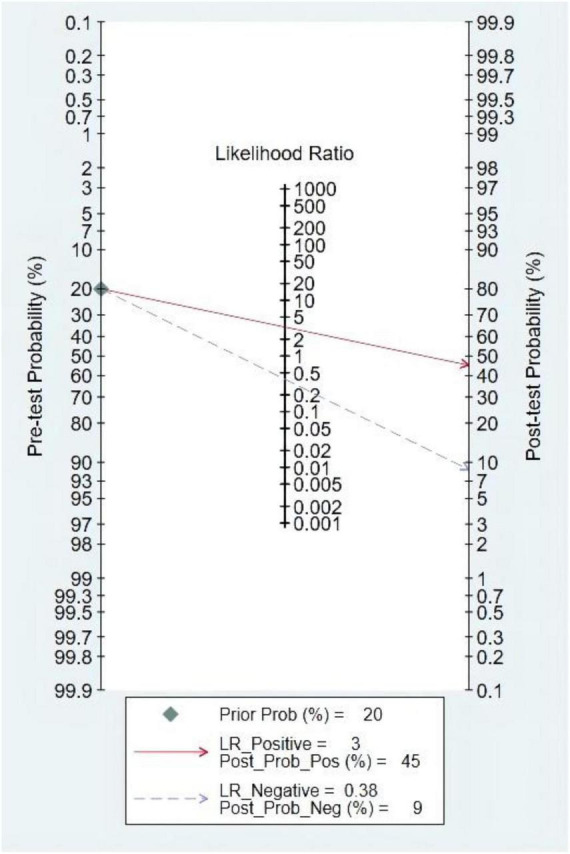
Fagan diagram of left ventricular ejection fraction (LVEF) in the diagnosis of heart sound.

The pre-test probability was 20%, and the probability of HF was 45%. In addition, the LRP was <10 (LRP = 3) and the LRN was >0.1 (LRN = 0.38), indicating that the diagnosis could be neither confirmed nor excluded. Their predictive value of LVEF in HF was also limited. The details are also shown in Figure 12.

### Meta-regression and subgroup analysis

**FIGURE 13:**
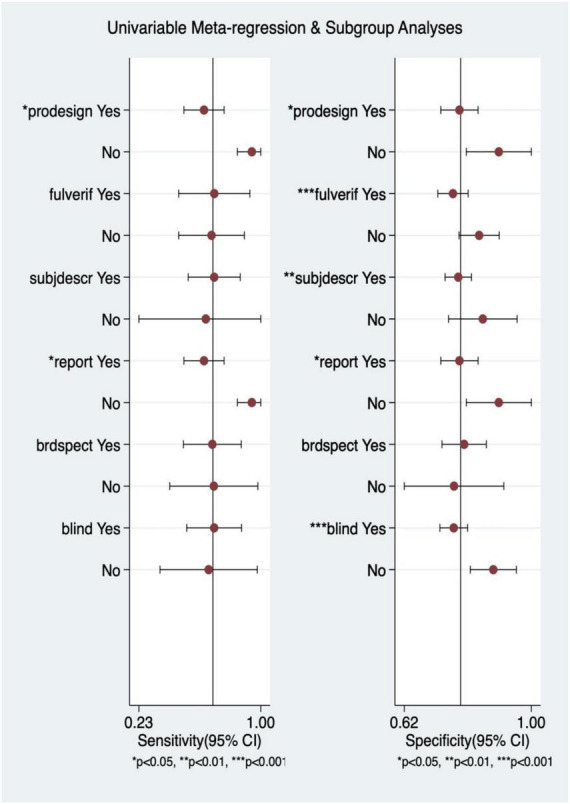
Multiple univariate meta-regression and subgroup analysis. Prospective design: prodesign; fulverif: partial verification bias; subjdescr: adequate description of study participants; brdspect: broad spectrum of disease.

Among the LVEF studies, the factors that might affect the heterogeneity, including prospective design (prodesign), partial verification bias (fulverif), an adequate description of study participants (subjdescr), report, a broad spectrum of disease (brdspect), and whether the test results were evaluated by a blind method, were evaluated. The meta-regression analysis of the aforementioned factors revealed that although the sources of heterogeneity of specificity were statistically related to the prodesign, fuverif, and blind, the sources of heterogeneity of sensitivity were not related to these factors, as shown in Figure 13.

### Comparison of third heart sound and left ventricular ejection fraction

Third heart sound and LVEF were compared using SROC, sensitivity, and specificity analysis. Among them, the predictive value of LVEF was better. The details are shown in Table 2.

**TABLE 2:** Diagnostic performance of third heart sound (S3) and left ventricular ejection fraction (LVEF).

Indicator	Sensitivity	Specificity	AUC	Sensitivity combined	Specificity combined	Prior *P*	PLR (%)	NLR (%)
LVEF	0.70	0.79	0.79	0.70	0.79	20	45	9
S3	0.23	0.94	0.49	0.23	0.94	20	48	17

## Discussion

Heart failure is a global public health issue of epidemic proportions and represents a tremendous burden to the overall healthcare costs (26). Meanwhile, it affects the quality of life of patients and their families seriously. Therefore, early recognition and accurate diagnosis are essential, and meaningful for a positive outcome.

This systematic review and meta-analysis was novel in comparing the ability to diagnose HF between S3 and LVEF. In this meta-analysis, 19 studies, including 5,614 participants, were analyzed. The combined sensitivity and DOR of S3 was less than that of LVEF. On the contrary, S3 had a higher specificity than LVEF, and the AUC of S3 was less than that of LVEF. Moreover, after using the S3 or LVEF, the post-test probability was equally improved. This suggested that LVEF had the highest diagnostic value compared with S3 and S3 alone was not of high diagnostic value for HF.

The 2021 ESC guidelines (5) pointed out that the identification of the etiology of the underlying cardiac dysfunction was imperative in the diagnosis of HF, making it convenient for subsequent treatment decision-making. In general, HF is due to systolic, diastolic, or both dysfunction. However, the pathology of the valves, pericardium, or endocardium and the abnormalities of heart rhythm, and conduction can contribute to HF (27). Heart sound intensity and frequency and their relationship or the occurrence of heart murmur are closely related to the condition of a cardiac valve, myocardial contraction, and blood flow in the heart (28). The aforementioned arguments laid the solid foundation for the diagnosis of HF *via* heart sound detection and analysis.

Kosmicki et al. (29) found that the diagnostic efficacy of the discriminative model constructed based on the characteristics of heart sound-electrocardiogram fusion, which included the S3 strength, left ventricular systolic time, electromechanical activation time, QR interval, and QRS interval, was significantly better than that of B-type natriuretic peptide (BNP). Moreover, the identification ability of patients with CHF in the “gray zone” of BNP significantly improved. Moreover, Maisel et al. (30) found that the strength of the S3 provided rapid results that assisted with the identification of acute HF (AHF) in selected populations. This evidence disclosed that S3 had high diagnostic value as an auxiliary diagnostic indicator. However, S3 was used as the single diagnostic indicator, reducing the sensitivity and specificity of diagnosis.

Meanwhile, the deficit in auscultation technology and the techniques of sound deciphering pulled down the sensitivity and specificity of diagnosis in HF *via* heart sound. The detection method for heart sound has been continuously improving, with new feature extraction algorithm and computer-aided diagnosis system based on machine learning or deep learning (31, 32), which further improves its diagnostic value. Liu et al. (31). reported that using extreme learning machine and heart sound characteristics to assist in diagnosing HF with preserved ejection fraction (HFpEF) showed an accuracy of 96.32%, a sensitivity of 95.48%, and a specificity of 97.10, which demonstrated the effectiveness of HS for HFpEF diagnosis. Alkhodari et al. (33) showed that the potential of implementing deep learning-based models clinically to ensure faster, yet accurate, automatic prediction of HF based on the ASE/EACVI LVEF guidelines with only clinical profiles, and corresponding information as input to the models. In addition, Alkhodari et al. (34) also found that applied support vector regression (SVR) models to estimate LVEF from ECG derived heart rate variability (HRV) data which ensure the best possible estimations of LVEF levels. Although the diagnostic value of S3 as a single indicator for HF is not high, heart sound shows promise as a diagnostic and prognostic tool in HF with the update of heart sound feature extraction methods.

## Conclusion

The use of S3 alone presented lower sensitivity in the diagnosis of HF compared with LVEF, whereas it was useful in early pathological assessment. Future prospective studies are needed to explore the diagnostic value of heart sound analysis based on new feature extraction algorithm and computer-aided diagnosis system based on machine learning or deep learning, so as to improve the early recognition rate of HF.

### Study limitations

First, less and reduplicative studies made the original data incomplete. Second, moderate heterogeneity existed across studies, and meta-regression and subgroup analyses failed output due to limited S3 data. Third, few included studies did not explicitly exclude participants. These shortcomings should be further investigated and addressed in future studies.

## Data availability statement

The original contributions presented in this study are included in the article/Supplementary material, further inquiries can be directed to the corresponding authors.

## Author contributions

LD, MH, and XL performed the literature screening, the data extraction and analysis, result representation, and drafting of the manuscript for intellectual content. FX performed statistical analysis, interpreted the data, and revised the manuscript for intellectual content. XW initiated the study and revised the manuscript for intellectual content. XF and CW revised the manuscript. All authors contributed to the study and approved the submitted version of the manuscript.
