# Supplementary material for: A systemic review and meta-analysis comparing the ability of diagnostic of the third heart sound and left ventricular ejection fraction in heart failure
Source: Front Cardiovasc Med. 2022 Oct 6;9:918051. doi: 10.3389/fcvm.2022.918051 (PMC9582155; doi:10.3389/fcvm.2022.918051)
Supplement: Supplementary file 1 [file Table_1.docx]

| **Supplementary Table 1. Inclusive and exclusive criteria for included studies** | | | | | |  |
| --- | --- | --- | --- | --- | --- | --- |
| **Author** | | **Inclusive criteria** | **Exclusive criteria** | **type of method used assess HF** | **founding** |  |
| Quyen Dao ^(9)^ | | Shortness of breath as a prominent complaint; associated symptoms could be edema, weight gain, cough, or wheezing | Patients whose dyspnea was clearly not secondary to CHF (trauma or cardiac tamponade); and patients with acute coronary syndromes | official reading of chest X-ray,  past history not available at the time for the emergency department physicians, the results of subsequent tests to measure systolic or diastolic function and, finally, the hospital course for patients admitted to the hospital. | B-type natriuretic peptide blood concentration measurement appears to be a sensitive and  specific test to diagnose CHF in urgent-care settings. |  |
|  | |  |  |  |  |  |
| Cathrine W. Knudsen ^(10)^ | | Sudden onset of dyspnea with no history of chronic dyspnea or an increase in the severity of chronic dyspnea | Patients with an obvious cause of dyspnea not due to heart failure (e.g., stabbing injuries, trauma, and pneumothorax) | radiographic evi dence of cardiomegaly/redistribution and BNP levels higher than 100 pg/mL as indicators of heart failure were assessed | In patients presenting to the emergency de partment with acute dyspnea, BNP levels and chest radiographs provide complementary diagnostic information that may be useful in the early evaluation of heart failure |  |
| Cathrine Wold Knudsen ^(11)^ | | All patients presented with shortness of breath as their main complaint. | Patients with chest pain ; patients in whom the shortness of breath was clearly not secondary to CHF (e.g. trauma) | All patients were examined by the medical resident or cardiology fellow on duty according to common clinical practice, and medical history and physical exam findings were recorded. A research fellow collected additional data, including current medication, electrocardiographic data and laboratory test results. | Point-of-care BNP measurement in the emergency department discriminates well between patients with dyspnoe of cardiac and non-cardiac origin regardless of age and gender |  |
| Zhang Wenbo ^(14)^ | | The echocardiographic examinations of patients with chronic heart failure were LVEF<50%, and all patients with heart failure received stable doses of anti-heart failure drugs; The normal control group is the healthy people who were hospitalized during the same period, and the echocardiography LVEF>50% | Patients with chronic atrial fibrillation, pre-excitation syndrome, and atrioventricular block; Patients with chronic obstructive pulmonary disease and valvular heart disease; Patients with congenital heart disease;Patients with severe liver and kidney disease | left ventricular end-diastolic diameter（LVEDd）, left ventricular eject fraction(LVEF) | the S3 strength proved to be useful in diagnosing chronic systolic heart failure |  |
| SEAN P. COLLINS ^(15)^ | | Patients were identified as potential participants if they were > 18 years of age, had an electrocardiogram (ECG) ordered, had signs or symptoms of heart failure (dyspnea, extremity edema, fatigue), and had provided written informed consent; patients with pacemakers or in atrial fibrillation | An ECG performed and more than 1 h had passed since they received vasodilators or diuretics for acute heart failure | an electrocardiogram(ECG) ordered, had signs or symptoms of heart failure (dyspnea,extremity edema, fatigue) | An S3 is highly specific for primary HF and it is ideally suited for use in combination with BNP to improve diagnostic accuracy in ED patients with dyspnea of unclear etiology |  |
| SEAN P. COLLINS ^(16)^ | | Eligible patients were at least 40 years of age, with dyspnea as a chief complaint | Patients with dialysis-dependent renal failure or whose dyspnea was clearly not related to acute decompensated heart failure (e.g., penetrating chest injury or blunt trauma) | Laboratory tests, chest radiography findings, demographics, medical  history, physical examination, and ECG finding | the acoustic cardiography S3 was specific for acute decompensated heart failure and affected physician confidence but did not improve diagnostic accuracy for acute decompensated heart failure |  |
| Shang Wang ^(17)^ | | Heart failure patients with LVEF <50% and LVEF ≥50% | Exclusion criteria for heart failure cohort included age 18 years, systolic blood pressure 90 mm Hg, mitral stenosis, constrictive pericarditis, use of mechanical ventilation, and pacemaker implantation; informed consent was obtained from each patient | echocardiographic findings, End-diastolic and end-systolic volumes, left ventricular ejection fraction (LVEF) | this bedside technology(including acoustic cardiographic parameters in cluded S3 score (probability that the third heart sound exists), Receiver operative characteristic curves were used to determine diagnostic utility of acoustic cardiography.) may be helpful in identifying HF |  |
|  |  |  |  | the Framingham score for HF plus echocardio graphic evidence of systolic dysfunction |  |  |
| B Dieplinger ^(18)^ | | Patients with dyspnea as a chief complaint | NA | sonography of the IVC and aorta by an investigator.  Laboratory, radiographic, and clinical outcomes were  compiled during each patient's ED stay and throughout  any admission to the hospital. | only increased BNP and MR proANP concentrations remained independent markers for  the diagnosis of HF |  |
| Joseph B. Miller MD ^(19)^ | | Adults 50 years and older with the chief complaint of dyspnea | Mechanical ventilation, trauma, known portal hypertension, abdominal surgery in the last 14 days, and pregnancy | Acoustic cardiography; Echocardiography | the use of the  bedside caval index to provide hemodynamic estimates in diagnosing AHF. |  |
| Shang Wang ^(20)^ | | NA | Age <18 years, systolic blood pressure <90 mm Hg, severe mitral stenosis, constrictive pericarditis, and use of mechanical ventilation and pacemaker implantation | results of further cardiac tests such as Doppler echocardiography, cardiac catheter ization, radionuclide ventriculography, and pulmonary functional tests. | S3 score obtained by acoustic cardiography identified HFREF patients with severely im pairedsystolic and diastolic function, respectively |  |
| Damien Logeart, MD ^(21)^ | | All patients presenting to the emergency room of our institution for acute severe dyspnea | Patients with acute myocardial infarction, chest injury, or recent surgery | results of physical examination and other blood tests, and interpretations of chest radiographs or other diagnostic tests were collected. | BNP measurement and Doppler echocardiography are both useful for establishing the cause of acute dyspnea |  |
| Philippe Gabriel Steg, MD ^(22)^ | | Patients had to have shortness of breath as their most prominent complaint | Patients aged 18 years, those whose dyspnea was not secondary to CHF (e.g., cardiac tamponade), and patients with acute myocardial infarction, renal failure, and obvious trauma were excluded; patients with unstable angina were excluded unless their predominant presentation was dyspnea | eft ven tricular (LV) ejection fraction and pulsed Doppler  analysis of mitral | combined BNP measurement echocardiographic determination of EF have marked additivediagnostic value. |  |
| Peiman Nazerian ^(23)^ | | All adult patients presenting for dyspnea, as their main symptom, while an investigator was present, including acute-onset dyspnea or worsening of chronic dyspnea at the time of the treating physician evaluation | Patients with trauma, ST-elevation myocardial infarctions, or dyspnea clearly caused by something other than heart failure, such as pneumothorax; patients who had received intravenous (IV) therapy in the emergency department (ED), before EDecho and NT-proBNP were performed; if the investigator judged that both left ventricular ejection fraction and pulsed Doppler analysis of mitral inflow were not interpretable due to a very poor acoustic window | Participants were identified by the treating physician, a brain natriuretic peptide (BNP) | EDecho, particularly pulsed Doppler analysis of mitral inflow, is a rapid and accurate diag nostic tool in the evaluation of patients with acute dyspnea. |  |
| Kenton L. Anderson MD ^(24)^ | | Adult patients presented to the ED with acute dyspnea and if the treating attending physician felt that CHF exacerbation was part of the differential diagnosis after the history and physical examination but before any testing was completed | Patients were younger than 18 years or unable to consent | all enrolled patients received conventional physical examination, rapid lung, cardiac, and inferior vena cava [IVC] ultrasound with a hand-held device (VscanW), electrocardiography, blood tests (including brain natriuretic peptide assay), and chest X-ray in the emergency room. | Ultrasonographic evaluations was 100% specific for the dx of ADHF |  |
| Katsuya Kajimoto ^(25)^ | | Patients admitted to the ED with acute dyspnea | Patients with acute coronary syndrome or chest injury; and patients who had acute dyspnea due to neither cardiac nor pulmonary cause | Echocardiography | rapid evaluation by LCI integrated ultrasound is extremely accurate for differentiating acute dyspnea due to AHFS from that caused by primary pulmonary disease in the emergency setting. |  |
| Ping Hu ^(26)^ | | Patients with chronic heart failure associated with dyspnea, palpitations, and fatigue symptoms | Patients with organ diseases; patients with hematological diseases and malignant tumors; patients with low compliance; and patients with lactation and pregnancy | ECG, Echocardiography | Echocardiography is a common method for the detection of chronic heart failure with multiple etiologies. |  |
| Hui Jiang ^(30)^ | | Patients with suspected chronic heart failure admitted to Shangrao Municipal Hospital | NA | Doppler echocardiography, cardiac catheter ization, radionuclide ventriculography, and pulmonary functional tests | The combined diagnostic value of echocardiography and BNP level detection is higher |  |
| Damien Logeart ^(21)^ | | Cause of acute dyspnea was assessed by the senior physician attending the patient on admission, on the basis of a physical examination, electrocardiogram, and chest x-ray examination | Patients with acute myocardial infarction, chest injury, or recent surgery | All patients underwent lung ultrasound examinations, along with basic laboratory testing, rapid NT-proBNP testing and chest X-rays. | BNP measurement and Doppler echocardiography are both useful for establishing the cause of acute dyspnea |  |
|  |  |  | Patients were subsequently excluded because of treatments (mechanical ventilation, diuretics, nitrates, or inotropic agents) started more than 2 h before arrival in our department, or because emergency echocardiography was not feasible | Echocardiographic determination of ejection fraction (EF) and point-of care BNP measurement for the diagnosis of CHF. |  |  |
| Philippe Gabriel Steg ^(22)^ | | Patients had to have shortness of breath as their most prominent complaint | Patients aged 18 years, those whose dyspnea was not secondary to CHF (e.g., cardiac tamponade), and patients with acute myocardial infarction, renal failure, and obvious trauma were excluded; patients with unstable angina were excluded unless their predominant presentation was dyspnea | radiographic evi dence of cardiomegaly/redistribution and BNP levels higher than 100 pg/mL as indicators of heart failure were assessed | combined BNP measurement echocardiographic determination of EF have marked additivediagnostic value. |  |
| Abbreviations: NA, not available from original study paper or supplementary or registration information; LVEF, left ventricular ejection fraction; S3, the third heart sound; ED, emergency department; AHFS: acute heart failure. | | | | | |  |
|  |  | | | | |  |
|  |  |  |  |  |  |  |
