# Supplementary figures and images for: A systemic review and meta-analysis comparing the ability of diagnostic of the third heart sound and left ventricular ejection fraction in heart failure
Source: Front Cardiovasc Med. 2022 Oct 6;9:918051. doi: 10.3389/fcvm.2022.918051 (PMC9582155; doi:10.3389/fcvm.2022.918051)

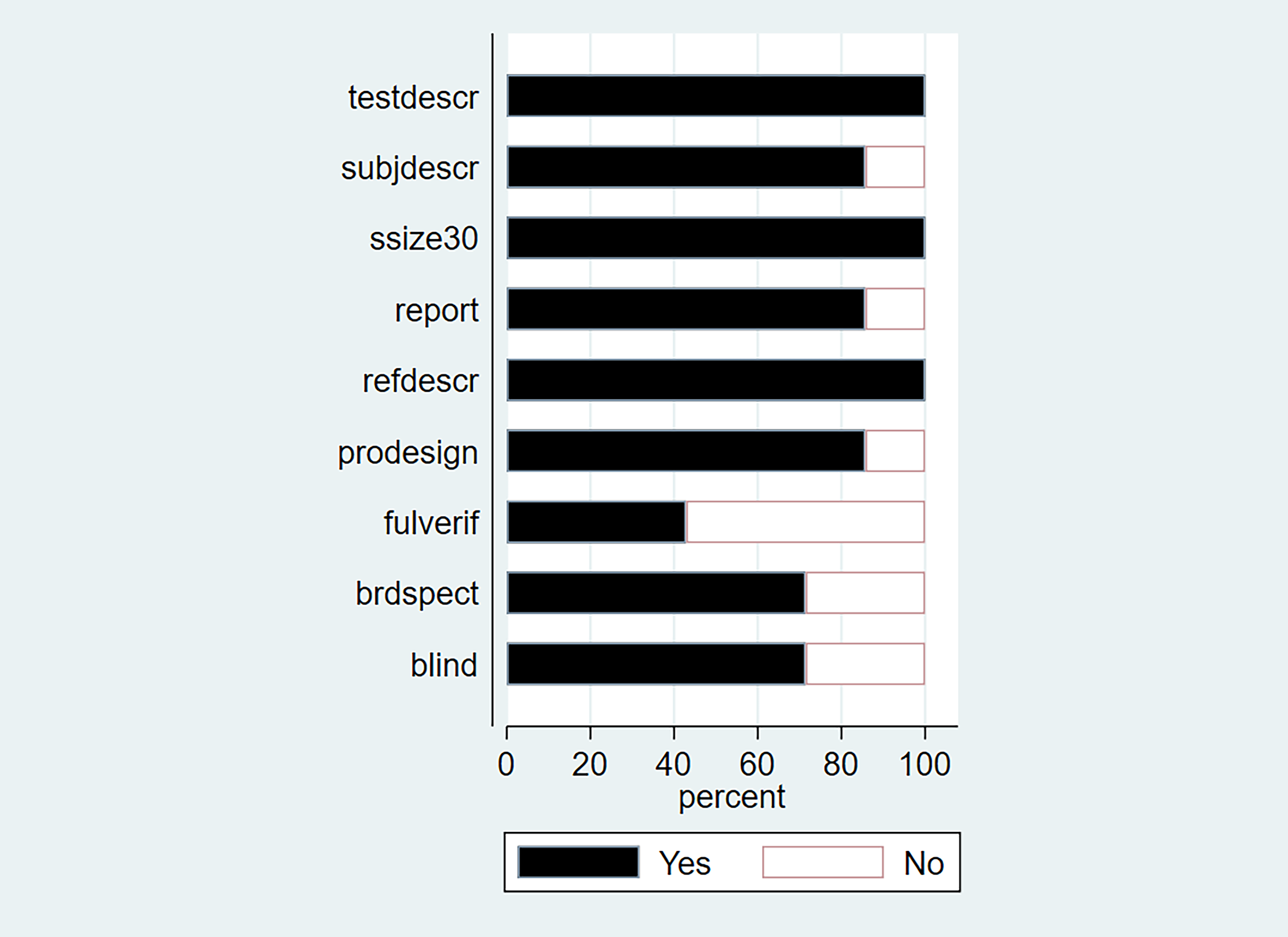

Supplement: Supplementary file 2 [file Image_1.TIF]

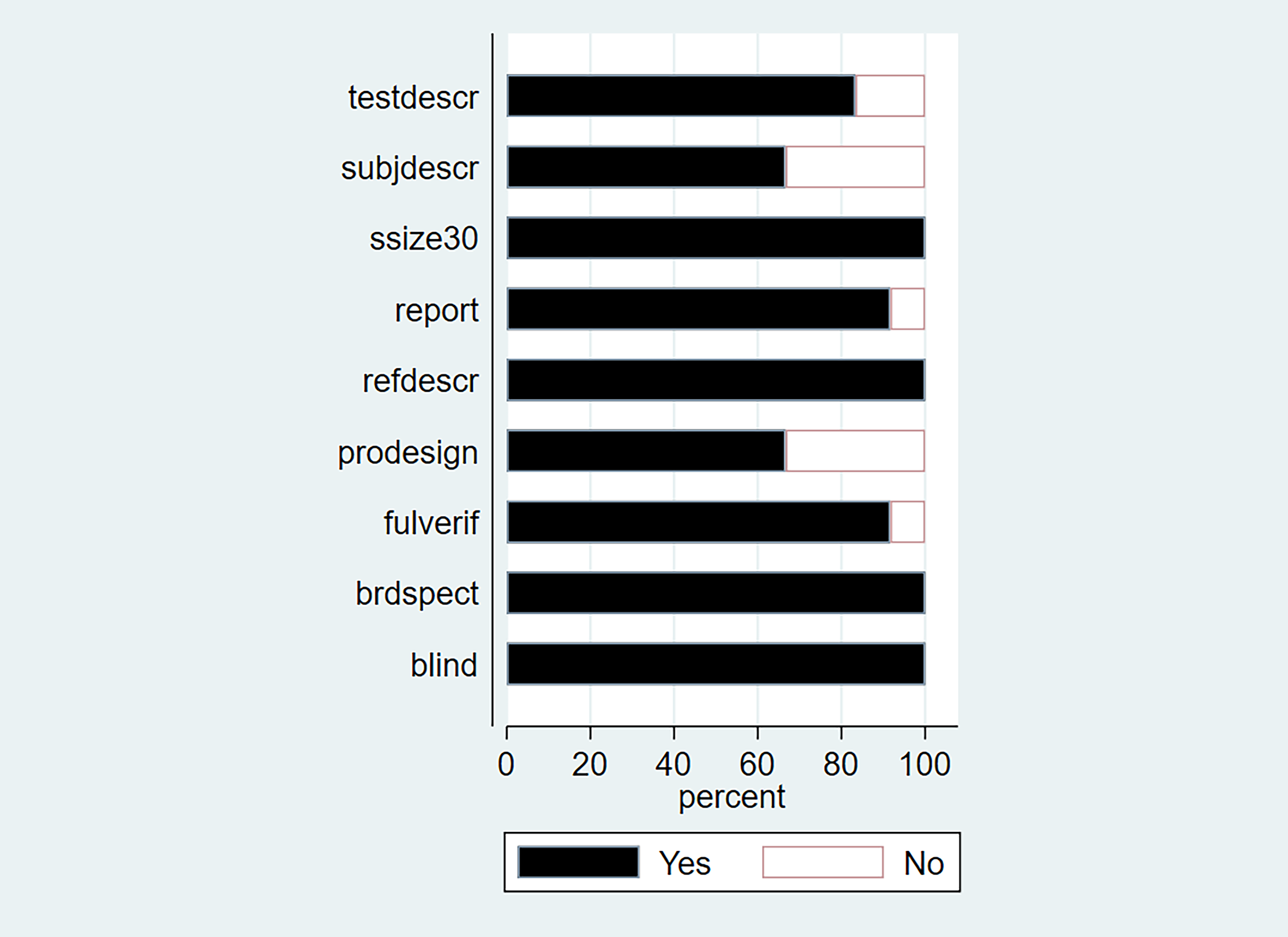

Supplement: Supplementary file 3 [file Image_2.TIF]
